# The association between restricted activity and patient outcomes in older adults: systematic literature review and meta-analysis

**DOI:** 10.1186/s12877-024-04866-w

**Published:** 2024-04-04

**Authors:** Ishbel L. Henderson, Rory W. Bone, Richard Stevens, Rebecca K. Barnes, Nia Roberts, James P. Sheppard, Richard J. McManus

**Affiliations:** 1 Nuffield Department of Primary Care Health Sciences, University of Oxford, Oxford, OX2 6GG UK; 2 NHS 24, NHS Scotland, Glasgow, G51 4EB Scotland

**Keywords:** Multimorbidity, Older adults, Restricted activity, Systematic review

## Abstract

**Background:**

Restricted activity is a potential early marker of declining health in older adults. Previous studies of this association with patient outcomes have been inconclusive. This review aimed to evaluate the extent to which restricted activity is associated with decline in health.

**Methods:**

A search was conducted for studies including people over 65 years old which investigated the association between measures of restricted activity and hospitalisation, cognitive decline, and mortality. Following data extraction by two reviewers, eligible studies were summarised using Inverse Variance Heterogeneity meta-analysis.

**Results:**

The search identified 8,434 unique publications, with 11 eligible studies. Three measures of restricted activity were identified: bed rest, restricted movement, and dependency for activities of daily living (ADL). Three studies looked at hospitalisations, with two finding a significant association with bed rest or restricted movement and one showing no evidence of an association. Restricted activity was associated with a significant increase in mortality across all three measures (bed rest odds ratio [OR] 6.34, 95%CI 2.51–16.02, I2 = 76%; restricted movement OR 5.38 95%CI 2.60–11.13, I2 = 69%; general ADL dependency OR 4.65 95%CI 2.25–9.26, I2 = 84%). The significant heterogeneity observed could not be explained by restricting the analysis by length of follow-up, or measure of restricted activity. No meta-analysis was conducted on the limited evidence for cognitive decline outcomes.

**Conclusions:**

Limited studies have considered the prognostic value of restricted activity in terms of predicting future declining health. Current evidence suggests restricted activity is associated with hospitalisation and mortality, and therefore could identify a group for whom early intervention might be possible.

**Supplementary Information:**

The online version contains supplementary material available at 10.1186/s12877-024-04866-w.

## Introduction

Worldwide, the prevalence of multiple long-term conditions (MLTC) is increasing [1]. These can include chronic physical conditions, non-communicable diseases, mental health conditions, and/or infectious diseases with long durations [1]. MLTC tend to accumulate over time, posing particular challenges for older people, who are already more vulnerable to poorer health outcomes [2]. The burden of morbidity associated with MLTC also contributes significantly to the workload in primary and secondary care settings [3].

One key challenge is identifying declining or deteriorating health. A disease-specific approach may lead to missed opportunities for intervention, as the responsibility for care falls between different specialties or institutions. This can lead to duplication of efforts, increased patient burden, and potential clashes between multiple treatment and monitoring approaches. Avoiding hospitalisation is a priority for patients [4], so it is crucial to identify markers that enable early intervention. Increasing MLTCs are associated with an increased risk of hospitalisation and death [5] and general reduced quality of life. Patients with MLTCs could benefit from having a holistic measure to identify generic decline before it presents acutely. Having a general measure of health could be useful for older people, many of whom might otherwise be unable to self-monitor their conditions.

Restricted activity, broadly defined as a reduction in a person's usual activities, ultimately leading to being unable to get out of bed could be suitable as such a measure. Previous studies have indicated an association between restricted activity and higher rates of hospitalisation [6] and mortality [7–9], as well as a reciprocal relationship with cognitive decline [10]. These studies suggest that such changes can occur several months before the outcome event of interest, indicating the potential for early intervention [8]. Although, the extent of this association varies widely between studies.

The aim of this review was therefore to examine the extent to which different measures of restricted activity are associated with all-cause hospitalisation, all-cause mortality, and cognitive decline in older people.

## Methods

The protocol was developed and registered on Prospero prior to conducting the search (CRD42022315789) and the results reported following the Preferred Reporting Items for Systematic Reviews and Meta-Analyses (PRISMA) guidelines [11].

The review question was formed using the PICOS framework, with patient and public involvement (PPI) to ensure the outcomes selected were meaningful to the target patient group.

- *Population*: Older adults, above an average (mean or median) age of 65 + years old.
- *Intervention* (exposure): Restricted Activities of daily living (increasing), with no disease specific cause
- *Control*: No restricted Activities of daily living (or decreasing activities of daily living)
- *Outcome*: Hospitalisation, mortality, cognitive (changed Jun 2022) decline
- *Study design*: quantitative studies, specifically cohort studies, case control studies, or randomised control trials

### Search strategy and selection criteria

A search was conducted from inception to 28th May 2022, using five databases: MEDLINE (via Ovid), Embase (via Ovid), Web of Science, CINAHL, and ASSIA. Search terms were designed to capture studies including older adults which investigated the association of restricted activities, including activities of daily living, to subsequent clinical outcomes. No language limits were applied. Studies utilising disease specific or post-operative populations were excluded. A copy of the search can be found in the appendix (Supplementary Material 1: Appendix A).

### Inclusion criteria

- Studies including people with an average age of 65 years or over, measuring a reduction of usual activity
- Quantitative study designs including observational, cohort, case control, longitudinal, and interventional studies
- Measuring at least one relevant outcome from hospitalisation, mortality, and cognitive decline
- Primary data reported

### Exclusion criteria

- Disease specific studies, or post-operative studies (forced restricted activity)
- Secondary data, unless drawn from national statistical surveys
- Analyses which did not compare patients with restricted activity to an appropriate control group
- Literature reviews

### Outcomes

The primary outcome for this review was all-cause hospitalisation. Secondary outcomes of interest were functional decline and all-cause mortality. These outcomes were selected to identify generic decline in older adults. The outcomes were selected with the help of a patient and public involvement (PPI) group. During initial screening, it was identified that the outcome of functional decline was too similar to the exposure of restricted activity and so this was altered to cognitive decline. The search and inclusion criteria were updated accordingly and rerun.

### Data screening and extraction

Firstly, de-duplication was performed (using EndNote 20 (Clarivate)) and studies were screened by two independent reviewers using Rayyan (Rayyan.ai). Initial screening of title and abstracts excluded studies that clearly did not meet the inclusion criteria. If deemed relevant, or requiring further information, then full texts were independently screened for inclusion or exclusion. Any discrepancies were resolved either by discussion, or with an independent third reviewer.

Studies in different languages were initially screened using a translation software (DeepL Translator). If deemed potentially eligible, data were extracted by native speakers. Studies with relevant data missing were followed up with the corresponding author but none replied with relevant information. Where studies used data from the same cohort of patients, the most relevant paper using each dataset was included [6].

Following piloting, data extraction was conducted by two independent researchers. Extracted information included study and participant characteristics, methods of measuring activity and outcomes, which outcomes were investigated, statistical analysis, and results. Additional citation searching was performed on included papers to identify any relevant studies missed in the search.

A flowchart, following the PRISMA guidelines, was created to illustrate this process (Supplementary Material 1: Appendix B).

### Bias

The Quality In Prognosis Studies (QUIPS) risk of bias (RoB) tool [12] was used to assess the risk of bias across the studies. Two independent researchers conducted the RoB, with any conflicts resolved through a third reviewer.

### Data synthesis

Results were grouped by type of restricted activity. When individual activities of daily living (ADL) dependence were reported separately, the most relevant measures were extracted: walking, transferring, and dressing. Walking was grouped with restricted movement, while transferring and dressing were investigated separately. A post-hoc analysis pooled studies based on their follow-up periods; short follow up, between 12–24 months, and 25 + months. Within each group, the results were ordered by those with adjusted data and non-adjusted data. No overall meta-analysis across all studies was undertaken due to differences in the way restricted activity was measured.

To reduce heterogeneity, the data describing the most ‘conservative’ definition of restricted activity (e.g. fully dependent for an activity, rather than partially dependent) was used, if different levels were reported. A sensitivity analysis was conducted varying the definitions for restricted activity (e.g. fully dependent and partially dependent for an activity, see Supplementary Material 1: Appendix C).

Meta-analyses were conducted using the using the Inverse Variance Heterogeneity method [13], which combines the weighting scheme of the fixed effects model with the variance estimation of the random effects model. This was done in STATA (version 16.1), using the admetan command [14]. Results were displayed using forest plots. Odds ratios were extracted were possible; if not available, risk ratios (RR) or incident rate ratios (IRR) were extracted instead. When analysing rare outcomes, these are approximately equivalent. To address confounding, we extracted adjusted odds ratios adjusted for confounders where available. Heterogeneity was summarised using the I^2^ statistic.

## Results

Initial searches resulted in 9,181 studies reduced to 8,434 following de-duplication. Following review of titles and abstracts, 42 full text articles were reviewed in detail for relevance and a total of 15 studies fulfilled the inclusion criteria (Supplementary Material 1: Appendix B). All included studies were observational cohort studies, designed to measure the association between restricted activity and clinical outcomes in real-world settings.

Included studies examined older adults, with mean ages ranging from 70 to 92 years. Population characteristics (Table 1) varied by ethnicity (90 to 40% white), with many unreported characteristics. Eight studies were conducted in the US [7, 15–20], and the others spread between Europe (Spain and Finland) [21, 22], Asia (Japan) [23], South America (Brazil) [24], and the Middle East (Israel) [9].

**Table 1:** Study population for the included studies

First Author and year	Study population Country	Average age	Number of participants	Females (%)	Ethnicity (White %)	Living alone (%)	Follow up period (months)	Measures of restricted activity	Outcome measure/s
Brill, 1999 [18]	Community-dwelling White or African American women, **United States**	74	24,612	100	90.3	Unknown, (42% married)	60	Restricted movement (Categorising participants into groups, dependant on how they perform the major activity)	Mortality
Clark, 1990 [7]	Independently ambulating individuals in assisted apartments, aged 65 or over who spent time in an intermediate-care facility at any time during the year, **United States**	84.5	297	82	Unknown	Unknown	12	Bed rest	Mortality
Gill, 2001 [6]	Nondisabled community-dwelling members of a large health plan, 70 years of age or older, New Haven, Connecticut, **United States**	78.4	754	64.4	90.5	39.5	12	Bed rest	Hospitalisations
Cabrera, 2012 [24]	Community-dwelling dependent older persons (age 60 and older), **Brazil**	77.5	130	69.2	Unknown	Unknown	12	Restricted movement and bed rest (Categorising participants based on their mobility patterns: those who could walk independently, those who walked with the help of caregivers or with the use of walking aids, those who were bedridden or confined to a wheelchair)	Mortality and hospitalisation
Zhao, 1993 [23]	Community-dwelling frail elderly people in Yao City, **Japan**	80.8	423	61	Unknown	62	12 + 60	Activities of daily living	Mortality
Carey, 2008 [15]	Community-dwelling subjects of All-Inclusive Care for the Eldery (PACE), Development cohort, **United States**	79	2,232	67.7	39.9	Unknown	36	Activities of daily living	Mortality
Carey, 2004 [16]	Community-dwelling subjects aged 70 or over, in the **United States**	78	4,516	61.0	84	Unknown	24	Activities of daily living	Mortality
Jylha, 1999 [21]	Community-dwelling people aged 90 or over in city of Tampere, **Finland**	92	366	81.1	Unknown	71.2	18	Restricted movement and bed rest (Questionnaire, asking how participants spend most of their day: on feet, sitting, or in bed)	Mortality
Ginsberg, 1999 [9]	Community-dwelling Jerusalem residents > 65 years, **Israel**	70	605	48	Mixed, unknown	Unknown	72	Activities of daily living and bed rest (days in bed per fortnight)	Mortality
Palomo, 2000 [22]	Primary care and community-dwelling, **Spain**	80	223	76.2	Unknown	Unknown	24	Activities of daily living and (hours in bed per day)	Mortality
Hardy, 2011 [25]	Community-dwelling adults aged 65 years or older enrolled in Medicare, **United States**	76	5,895	57	81	Unknown, (29.2% married)	16	Restricted movement (ability to walk ¼ mile)	Mortality and hospitalisation
Rajan, 2015 [20]	Community-dwelling participants over the age of 65 with no functional limitations, in **Chicago United States**	70.7	3,825	53	Unknown, 65% African Americans	Unknown, 59% married	108	Restricted movement, measured by 8 Rosow-Breslau and Nagi limitations (ROS-B/Nagi)	Cognitive decline
Rajan, 2013 [19]	Community-dwelling participants over the age of 65 with no functional limitations, in **Chicago United States**	70.7	6,678	60.6	Unknown, 63.6% black	Unknown	36	Activities of daily living	Cognitve decline

Three studies examined outcomes relating to hospitalisation, and ten related to mortality (two measured both outcomes). Two studies examined outcomes related to cognitive decline. Follow-up periods ranged from 12 to 72 months.

### Measures of restricted activity

The measures used in included studies were bed rest (*n* = 6), restricted movement (*n* = 6), and activities of daily living (*n* = 4). These measures are evaluated in more detail in Supplementary Material 1: Appendix D.

Measures ranged from hours in bed, to walking across a room. All were simple questions a patient could report, through a range of methods: self-completed surveys [21], telephone interviews [6], and in-person interviews with a researcher [18, 24] or a healthcare professional [22]. Time-scales of follow-up periods varied from monthly [6] to annual [18, 24], or biennial [22] check-ups, over different follow-up periods.

The only measure that followed an established matrix was the ADL scale using the Katz index of independence [26] (Supplementary Material 1: Appendix D). This is scored on a 6-point scale, to determine physical functions and dependence (maintaining personal hygiene, transferring, ambulating, and feeding themselves). It was either reported as a combined score of all the measures [9, 16, 19, 22, 23], or dependency for each measure was reported individually [15, 16, 24]. For this study, dependency for walking was chosen as the most relevant ADL measure to explore further.

Other measures included how the respondent spent most of their day (e.g. sitting or in bed) [21], bed-bound states (measured by days or hours in bed [6, 9, 22]), and a combination of everyday activities (measured with 3-point Rosow-Breslau [27]) and limb strength (measured with 5-point Nagi measures [28]) used to create a Rosow-Breslau/Nagi measure [20] (Supplementary Material 1: Appendix D: evaluation of measures).

## Risk of bias

All papers had moderate risk of bias for at least two out of six sections (Table 2). Over half the papers had high risk of bias for one section, mostly due to potential study confounding, or study attrition rates. Taken together, there was an overall medium–high risk of bias, due to the high bias in study confounding, and statistical analysis sections. There was some uncertainty about methods used for missing data and missing confounder data, as most studies did not report this.

**Table 2:**
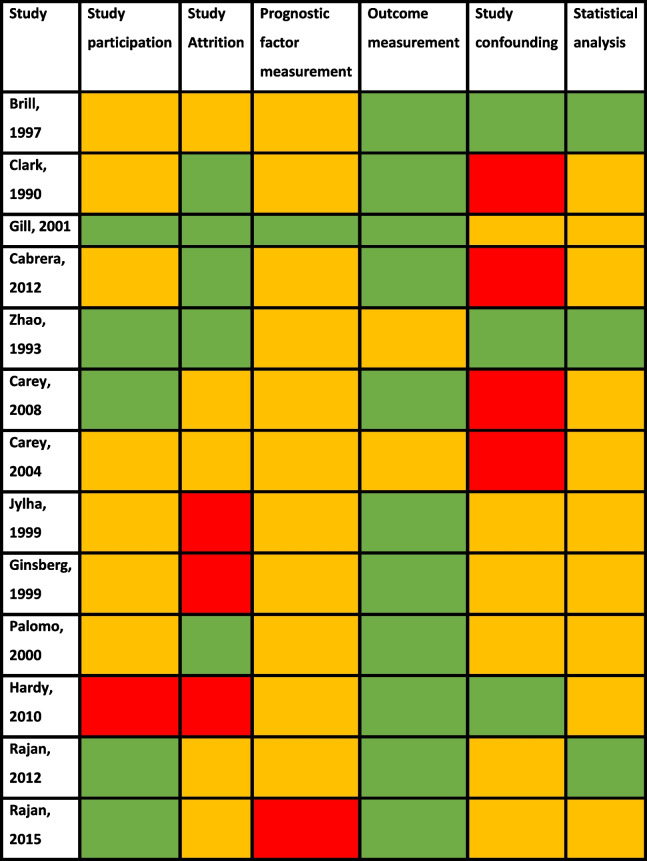
QUIPS risk of bias assessment (RoB). RoB was assessed following the QUIPS framework, and reported as low risk (green), moderate risk (orange), and high risk (red) [6, 7, 9, 15, 16, 18, 20–24, 29]

The main concerns highlighted by the risk of bias assessment were the lack of adjustment for confounding factors during analysis, where more than half of the studies didn’t make appropriate adjustments or models, and the prognostic factor measurement, which caused high heterogeneity. There were also some missing data from the patient characteristics, specifically for education and living situations, precluding assessment of whether living alone impacted the results.

This bias assessment also emphasised that the outcome measures had clear endpoints (mortality or hospital admissions), so the non-blinded design of the studies was not considered to bias the results.

### Primary outcome hospitalisation

**Fig. 1:**
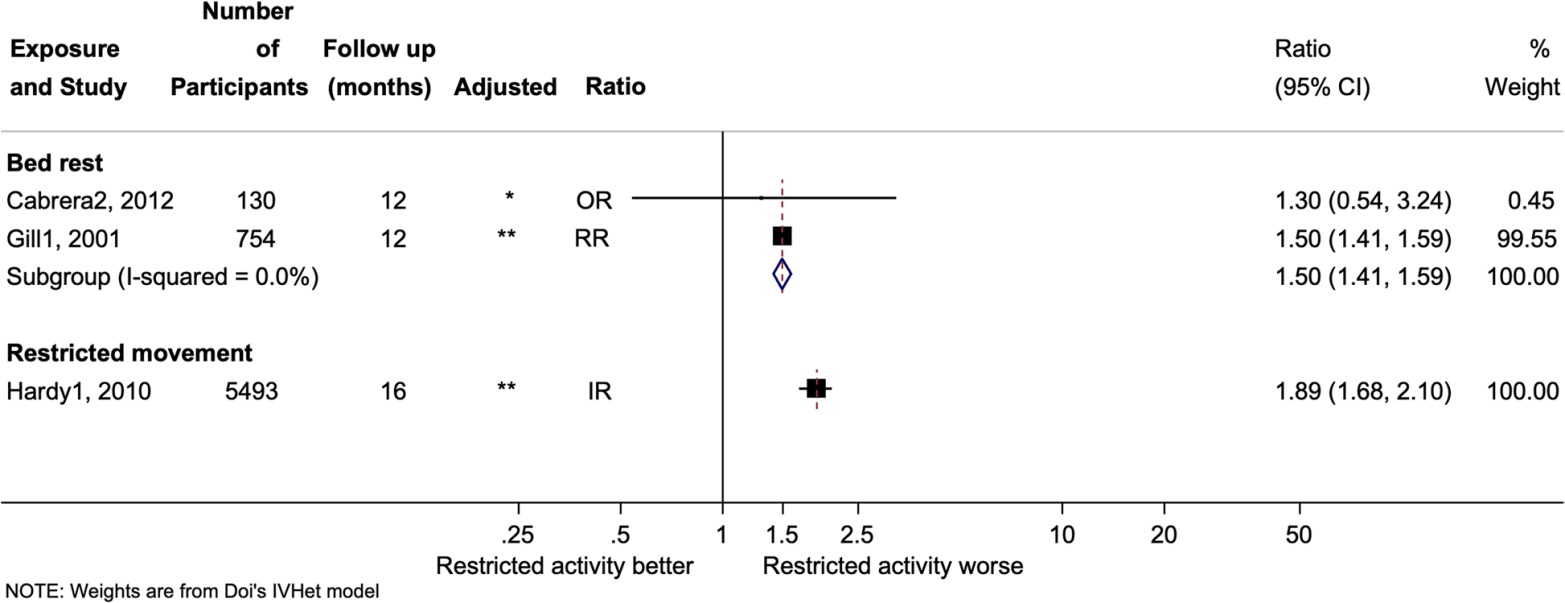
Forest plot of restricted activity and hospitalisation. Analysis is sub-grouped by the type of restricted activity (bed rest and restricted movement). The square markers indicate the point estimate of the effect size within the different studies, with the whiskers indicating the confidence intervals. The size of the box correlates to the inverse variance of the effect estimate, which indicates the weight given to the study in the pooled analyses. Superscript denotes the control for each study. RR = Risk ratio, OR = Odds ratio, IR = Incident rate. *No pooled effect was estimated due to the lack of studies in each sub-group, and due to the non-significant study sizes

Only three studies examined the association between restricted activity and hospitalisation (Fig. 1), with two studies measuring bed rest, and one examining restricted movement. Due to the methodological diversity of the small number of studies, combining data within a meta-analysis was deemed inappropriate. The association between bed rest and hospitalisations was similar in both studies, with a risk ratio of 1.30 (95% CI 0.54 to 3.24) [6] and 1.50 (95% CI 1.41 to 1.59) [24]. The other study found an association for restricted movement and hospitalisation (incident ratio (IR) 1.89, 95% CI 1.68 to 2.10) [25].

### Secondary outcomes

**Fig. 2:**
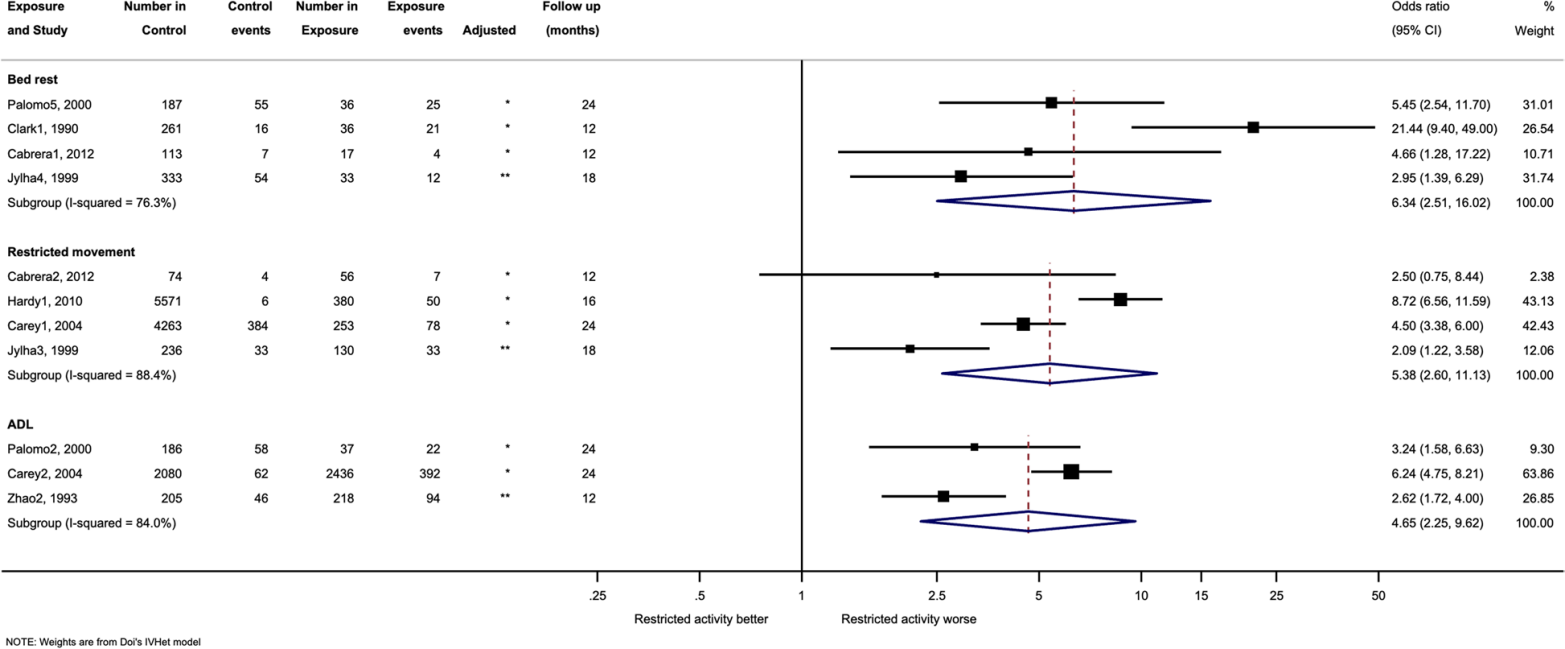
Forest plot and meta-analysis of restricted activity and mortality, with a short follow up (≤ 24). Analysis is sub-grouped by the type of restricted activity (bed rest, restricted movement, and activities of daily living (ADL) dependency). Square markers indicate the point estimate of the effect size within the different studies, with the whiskers indicating the confidence intervals. The size of the box correlates to the inverse variance of the effect estimate, which indicates the weight given to the study in the pooled analyses. The diamond markers indicate the pooled effect estimate across sub-groups. Superscript denotes the control for each study

**Fig. 3:**
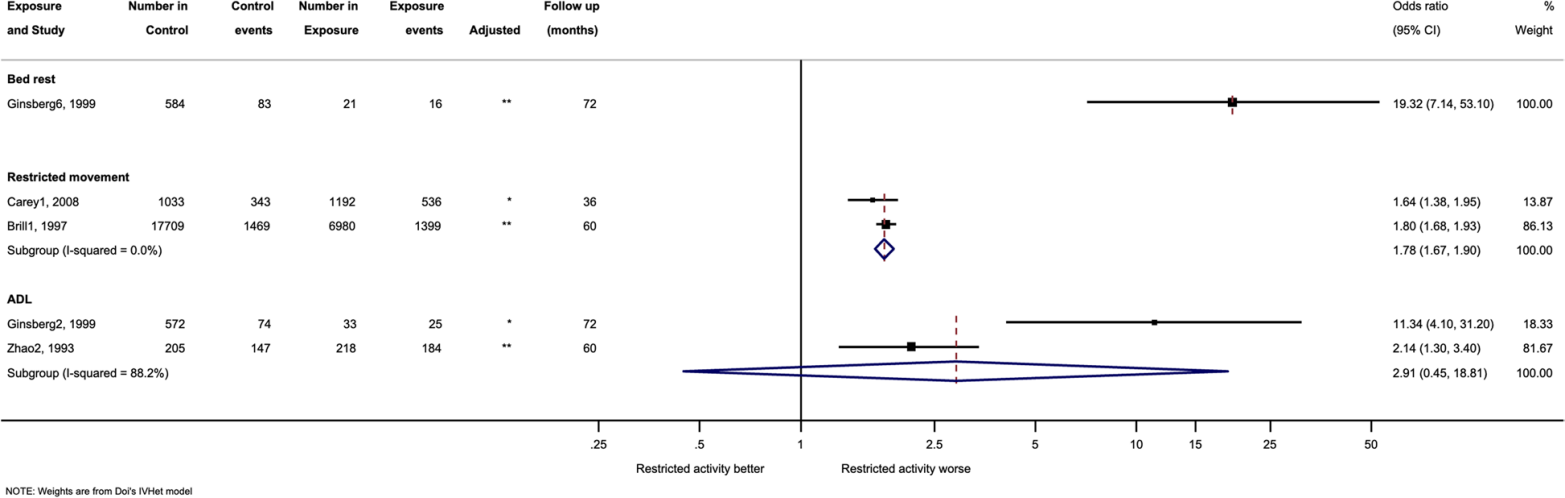
Forest plot and meta-analysis of restricted activity and mortality, with a long follow up (≥ 25). Analysis is sub-grouped by the type of restricted activity (bed rest, restricted movement, and activities of daily living (ADL) dependency). Square markers indicate the point estimate of the effect size within the different studies, with the whiskers indicating the confidence intervals. The size of the box correlates to the inverse variance of the effect estimate, which indicates the weight given to the study in the pooled analyses. The diamond markers indicate the pooled effect estimate across sub-groups. Superscript denotes the control for each study. * = Not adjusted. ** = Adjusted. ^1^ No cut-down in usual activity. ^2^Independent for ADL, or walking. ^3^Spending day moving around. ^4^ Spending day sitting or moving around. ^5^Less than the defined cut off (16 h) hours in bed. ^6^Less than the defined cut off (6 days) bed rest

A total of ten studies examined the association between restricted activity and mortality (Figs. 2 and 3). Included studies had differing levels of adjustment, from no adjustment at all, to adjusting for a few confounders such as age, gender, or education level. For studies in which adjustment included gait speed or ADL dependency the unadjusted ORs were used to avoid over-adjustment, noting that these factors may be mediators rather than confounders.

Analyses were split by those examining short-term follow-up (8 studies; up to 24 months) and those examining studies with longer term follow-up (4 studies; ≥ 24 months). In short-term studies, bed rest was associated with a sixfold increase in the risk of mortality (OR 6.34, 95% CI 2.51 to 16.02; I^2^ = 76%). The results were similar for restricted movement (OR 5.38 95% CI 2.60 to 11.13; I^2^ = 69%) and general ADL dependency (OR 4.65, 95% CI 2.25 to 9.62; I^2^ = 84%) (Fig. 2). There were fewer studies with longer follow-up, however these showed similar trends. Longer follow-up restricted movement had a 1.7-fold increase (OR 1.78, 95% CI 1.67, 1.90; I^2^ = 0%), although this was based upon two studies, weighted heavily in favour of one particular study. General ADL dependency (longer follow-up) was associated with a threefold increase in mortality (OR 2.91, 95% CI 0.45, 18.81; I^2^ = 88%). No meta-analysis was conducted for bed rest over a longer follow-up as there was only one study. Significant heterogeneity remained for both follow-up periods. Overall higher point estimates were observed over the shorter follow-up periods (Figs. 2 and 3).

The literature on cognitive decline was Ambiguous*, there was clear evidence suggesting functional decline and cognitive decline were associated and may occur simultaneously. The literature highlighted an important link between the two, however it was not clear which one preceded the other [30–32]. Most of the literature focused on cognitive decline impacting functional abilities [10, 29].

Two studies examined the effect of restricted activity on cognitive decline, by the same author with a crossover of participants, from The Chicago Health and Aging Project [33]. These papers showed restricted activity was associated with a 30% increase in cognitive decline compared to those without restricted activity, over a 9 year period [20], and that restricted activity was associated with accelerated cognitive decline over 3 years, of around a 158% increase compared to people without restricted activity [19]. Due to the insufficient number of studies, and the crossover of patients in the included studies, no meta-analysis was conducted.

Sensitivity analyses were* conducted, evaluating different definitions of restricted activity, which found similar directions of effect to the overall results but did not explain the statistical heterogeneity observed in the primary analyses (Supplementary Material 1: Appendix C).

## Discussion

### Summary of findings

This systematic review and meta-analysis has found some evidence of an association between restricted activity and hospitalisations, but data were sparse, precluding firm conclusions about the strength of any association. There was however evidence that restricted activity was associated with an increased risk of mortality. Given these links with poor subsequent outcomes, these findings support the hypothesis that restricted activity could be a potential tool to prospectively identify decline in older adults. Three measures of restricted activity were identified (bed rest, restricted movement, and general ADL dependency), all being administered through questionnaires.

### Strengths & limitations

To our knowledge, this is the first systematic review and meta-analysis to examine the association of restricted activity on patient hospitalisations, cognitive decline, and mortality.

Statistical heterogeneity was high within all measures examined and seems likely to be driven by a combination of the different populations and different definitions and measurements of restricted activity. There was also significant heterogeneity within the sub-groups, potentially due to different analytical approaches taken by individual studies. This should be considered when interpreting the results: in particular, the exact numerical estimates (odds ratios) should be considered less informative than the general finding.

There was also potentially high methodological heterogeneity between the studies, especially within control groups. Some studies used no change in activity as their control, whereas others compared restricted activity against partial limitations in activities. Some potentially relevant studies were also excluded due to not reporting any control group data.

The small number of available studies with evidence of low, and moderate risk of bias presented further limitations when interpreting the results. Unadjusted data have interpretation bias due to the potential impact of confounding factors. Similarly, when adjusted, studies did not always mention precisely which factors had been adjusted for [9]. Some studies over-adjusted for factors such as gait speed, which we considered potentially linked to progression of restricted activity. Despite this, the point estimates for all included studies showed a direction of effect towards restricted activity being associated with poorer outcomes. This was further confirmed by the sensitivity analysis evaluating partial restricted activity, showing similar trends, but with lower overall risks. This supports the theory that the worse the restriction of activity, the worse the health outcome.

There were also some limitations with the study. One limitation was using a minimum average age, rather than actual age. This was a pragmatic approach for screening, as many abstracts reported averages rather than ranges. The lowest limit of age in an included study was 60 years (mean age 74 years) [24], however the impact of this, if any, is that any observed associations may have been under-estimated due to potentially lower mortality in younger populations.

The search was designed to capture a compressive overview of the literature on this topic.

However, the search term for cognitive function was limited to cognitive decline and didn’t include other terms such as cognitive trajectories or specific cognitive impairments (eg Dementia or Alzheimer’s). This is because we were interested in generically identifying causes of decline. Further exploration into the relationship between cognitive function and restricted activity might be beneficial, but is beyond the scope of this review.

Another limitation of the search strategy was not including care or nursing home admissions or receiving at home care as outcomes of interest. Many older people experience illness or injury, resulting in a move to residential care [34]. However, it can also be hypothesised that many might have been hospitalised prior to institutionalisation. While a few papers assessed during screening indicated institutionalisation could be a relevant outcome, there could have been other papers not included in the search results. Similarly, as our search criteria excluded qualitative studies, we were not able to assess the feasibility or acceptability of these measures to patients or their caregivers.

### Comparison with existing literature

This review was initially inspired by a Gill paper ‘Taking to bed at the end of life’ looking at bed rest in end-of-life care [8]. Gill’s data highlighted a link between restricted activity and poor patient outcomes using monthly interviews. Unfortunately, a lack of control data precluded inclusion of that study, but the results were consistent with Gill’s findings, showing similar effects of bed rest on patient outcomes.

In the present analyses, bed rest appeared to have the strongest association with mortality, compared to restricted movement and ADL dependency. The ADL (Katz-index) has a significant evidence base [26], validating a well-rounded set of questions to establish physical dependence, which was included in many of the studies [9, 15, 16, 22, 23]. An important consideration is timing of intervening, and which measure of restricted activity might present earlier, and therefore be a better opportunity for early intervention. More research is needed to better understand the timelines of restricted activity prior to an adverse outcome; the meta-analyses highlighted the highest risk was during shorter follow-up periods. This suggests intervention should occur within 12-24 months of experiencing restricted activity, however it can be hypothesised earlier intervention will result in better patient outcomes.

Studies using mobility measures (eg Lifespace [35]) did not meet our review eligibility criteria due to the composite nature of the assessment (i.e. a continuum of mobility combining cognitive and physical functions, and psychosocial and environmental factors).

Level 1 of Lifespace (mobility from one’s bedroom to other areas of the house) was highly relevant to this study, however its association with patient outcomes was not reported independently, and therefore could not be included in analysis.

Strong associations have been seen between lower Lifespace scores and poorer patient outcomes, in older adults, specifically, hospitalisation [36, 37], mortality [38–41], and cognitive decline [42, 43]. Despite not being eligible, these associations further support the findings that restricted activity has prognostic significance.

Both ADL dependency, or Lifespace are research tools and not currently used clinically for predicting patient outcomes, however evidence from this review show a reduction in activity is often observed before poorer patient outcomes. Interestingly, in primary care, it is often patients who bring up the effects a condition has on their everyday life, rather than doctors [44], which suggests activity restriction could be a meaningful measure for self-reporting health status.

### Implications for clinical practice and research

This review has identified an association between restricted activity and future mortality. This association provides optimism for the prospect of creating a general measure of health for patients with complex needs, particularly those with MLTC. Such an initiative could facilitate the assessment of their non-specific symptoms and enhance the early detection of potential health deterioration. Relatively simple questions could be comfortably self-reported by a range of patients with limited health literacy. Modern technology, such as smartphones, could collect such data, and flag changes potentially alongside passive measures such as step counting. However, the paucity of data highlights the need for more research in this area.

Limited studies were found assessing cognitive impairment. Cognitive decline was suggested by our PPI group as an important outcome to patients, as well as featuring in the James Lind Alliance priority setting for multiple conditions in later life [4]. Despite this, limited studies were found assessing cognitive impairment. Those that did found restricted activity increased the risk of cognitive decline, however, both papers were by the same researchers, on the same cohort of patients. Further high-quality studies are therefore required. Some studies have explored tracking movement using sensors within a ‘smart’ home setting, with the long-term goal of detecting early cognitive decline [45]. The Intelligent Systems for Assessing Aging Changes (ISAAC), demonstrated successful tracking of activity changes, including total activity, night-time activity, and walking speeds, however, has yet to be used to assess the prognostic significance of these measures.

## Conclusion

This study identified a range of measures of restricted activity, from bed rest to walking across a room. The measures demonstrated similar effects on patient outcomes, indicating an association between restricted activity and poorer patient outcomes, specifically mortality, and possibly hospitalisations.

This review indicates restricted activity could be a good general measure of health and predictor of future health decline. Further research is needed to determine whether restricted activity could be successfully incorporated into a clinically relevant system leading to earlier interventions for older adults with MLTC.

### Key points

- Restricted activity has been assessed in several ways, including using measures of bed rest, restricted movement, and activities of daily living.
- Despite few studies having explored the association between such measures of restricted activity and hospitalisation, the evidence indicates a possible association.
- Evidence suggests restricted activity is also associated with an increased risk of subsequent mortality, suggesting it could be used as an early warning signal for health decline in older adults.

### Why does this matter?

Restricted activity could be a good general measure of health and predictor of future health decline, for older adults who are unable to monitor their numerous conditions. A better understanding of the clinical meaning of restricted activity will help advance geriatric research.

### Supplementary Information

**Supplementary Material 1.**

## Data Availability

No primary data was used in this review. Please see referenced paper for primary data source. All data included in this study is in the public domain and has been published elsewhere. The aggregated dataset analysed during the current study is available from the corresponding author on reasonable request.

## Abbreviations

ADL: Activities of Daily Living
CI: Confidence Interval
ISAAC: Intelligent Systems for Assessing Aging Changes
IR: Incident Ratio
MLTC: Multiple Long-Term Conditions
OR: Odds Ratio
PICOS: Patient or problem. Intervention or exposure. Comparison or control. Setting.
PPI: Patient and Public Involvement
PRISMA: Preferred Reporting Items for Systematic Reviews and Meta-Analyses
RoB: Risk of Bias
RR: Risk Ratio
QUIPS: Quality In Prognosis Studies
