# Supplementary material for: The association between restricted activity and patient outcomes in older adults: systematic literature review and meta-analysis
Source: BMC Geriatr. 2024 Apr 4;24:316. doi: 10.1186/s12877-024-04866-w (PMC10993524; doi:10.1186/s12877-024-04866-w)
Supplement: Supplementary file 1 — Supplementary Material 1. [file 12877_2024_4866_MOESM1_ESM.docx]

# Appendices

# Appendix A: Search strategy

**Table A1-5: Search strategy (formatted for the different databases)**

| Medline (Ovid MEDLINE® Epub Ahead of Print, In-Process & Other Non-Indexed Citations, Ovid MEDLINE® Daily and Ovid MEDLINE®) | | |
| --- | --- | --- |
| 1 | exp Aged/ | 3364157 |
| 2 | ("old age" or (old* adj2 (people or person* or adult* or men or women or male? or female?)) or elder? or elderly or "over 60*" or "over 65*" or "over 70*" or "over 75*" or "over 80*" or "over 85*" or "over 90*" or "over 95*" or sexagenarian* or septagenarian* or octogenarian* or nonogenarian*).ti,ab,kw. | 731906 |
| 3 | 1 or 2 | 3731796 |
| 4 | Bed Rest/ | 4034 |
| 5 | (bed rest or bedrest).ti,ab,kw. | 6552 |
| 6 | (bed adj5 (confine* or immob*)).ti,ab,kw. | 1288 |
| 7 | (bedridden or bed ridden).ti,ab,kw. | 2905 |
| 8 | (prolong* adj3 immobili?ation).ti,ab,kw. or immobili?ation.ti. | 16004 |
| 9 | restricted activit*.ti,ab,kw. | 600 |
| 10 | (chronic adj2 "limitation of activity").ti,ab,kw. | 3 |
| 11 | 4 or 5 or 6 or 7 or 8 or 9 or 10 | 28297 |
| 12 | Activities of Daily Living/ | 69386 |
| 13 | Frailty/ | 5833 |
| 14 | Physical Functional Performance/ | 2189 |
| 15 | (cognitive adj2 (decline or status or capacity or recovery or outcome?)).ti,ab,kw. | 127190 |
| 16 | (physical* adj3 (decline* or challenge*)).ti,ab,kw. | 4644 |
| 17 | exp Mortality/ | 413906 |
| 18 | (mortality or death* or fatalit* or fatal outcome?).ti,ab,kw. | 1630295 |
| 19 | exp Hospitalization/ | 273200 |
| 20 | (hospitali?ation? or (length adj2 stay) or ((patient? or hospital) adj2 (admission? or admitted))).ti,ab,kw. | 368486 |
| 21 | 12 or 13 or 14 or 15 or 16 or 17 or 18 or 19 or 20 | 2356324 |
| 22 | 3 and 11 and 21 | 1549 |

| Embase | | |
| --- | --- | --- |
| 1 | exp Aged/ | 3303407 |
| 2 | ("old age" or (old* adj2 (people or person* or adult* or men or women or male? or female?)) or elder? or elderly or "over 60*" or "over 65*" or "over 70*" or "over 75*" or "over 80*" or "over 85*" or "over 90*" or "over 95*" or sexagenarian* or septagenarian* or octogenarian* or nonogenarian*).ti,ab,kw. | 1063205 |
| 3 | 1 or 2 | 3855964 |
| 4 | Bed Rest/ | 8787 |
| 5 | (bed rest or bedrest).ti,ab,kw. | 8528 |
| 6 | (bed adj5 (confine* or immob*)).ti,ab,kw. | 1865 |
| 7 | (bedridden or bed ridden).ti,ab,kw. | 4320 |
| 8 | (prolong* adj3 immobili?ation).ti,ab,kw. or immobili?ation.ti. | 18335 |
| 9 | restricted activit*.ti,ab,kw. | 741 |
| 10 | (chronic adj2 "limitation of activity").ti,ab,kw. | 5 |
| 11 | 4 or 5 or 6 or 7 or 8 or 9 or 10 | 36572 |
| 12 | daily life activity/ | 100975 |
| 13 | Frailty/ | 18052 |
| 14 | cognitive status/ or mental performance/ | 82280 |
| 15 | (cognitive adj2 (decline or status or capacity or recovery or outcome?)).ti,ab,kw. | 184371 |
| 16 | (physical* adj3 (decline* or challenge*)).ti,ab,kw. | 6233 |
| 17 | exp Mortality/ | 1217855 |
| 18 | (mortality or death* or fatalit* or fatal outcome?).ti,ab,kw. | 2323705 |
| 19 | hospital admission/ or hospital readmission/ or hospitalization/ or "length of stay"/ | 833461 |
| 20 | (hospitali?ation? or (length adj2 stay) or ((patient? or hospital) adj2 (admission? or admitted))).ti,ab,kw. | 633203 |
| 21 | 12 or 13 or 14 or 15 or 16 or 17 or 18 or 19 or 20 | 3597985 |
| 22 | 3 and 11 and 21 | 2141 |
| 23 | conference*.pt. | 5082487 |
| 24 | 22 not 23 | 1725 |

| S14 | S3 AND S6 AND S13 | 491 |
| --- | --- | --- |
| S13 | S7 OR S8 OR S9 OR S10 OR S11 OR S12 | 631,042 |
| S12 | TI ( (hospitalization*or hospitalisation* or (length N2 stay) or ((patient* or hospital) N2 (admission* or admitted))) ) OR AB ( (hospitalization*or hospitalisation* or (length N2 stay) or ((patient* or hospital) N2 (admission* or admitted))) ) | 93,884 |
| S11 | (MH "Hospitalization+") | 112,002 |
| S10 | TI ( (mortality or death* or fatalit* or (fatal N1 outcome*)) ) OR AB ( (mortality or death* or fatalit* or (fatal N1 outcome*)) ) | 377,186 |
| S9 | (MH "Mortality+") | 78,989 |
| S8 | TI ( (cognitive N2 (decline or status or capacity or recovery or outcome*)) ) OR AB ( (congitive N2 (decline or status or capacity or recovery or outcome*)) ) OR TI ( (cognitive* N3 (decline* or challenge*)) ) OR AB ( (cognitive* N3 (decline* or challenge*)) ) | 50,440 |
| S7 | (MH "Activities of Daily Living") OR (MH "Frailty Syndrome") OR (MH "Functional Status") OR (MH "Geriatric Functional Assessment") | 63,274 |
| S6 | S4 OR S5 | 4,885 |
| S5 | TI ( (bed N1 rest) OR bedrest OR (bed N5 (confine* OR immobil*)) OR bedridden OR (bed N1 ridden) ) OR AB ( (bed N1 rest) OR bedrest OR (bed N5 (confine* OR immobil*)) OR bedridden OR (bed N1 ridden) ) OR TI ( immobilisation or immobilization ) OR AB ( (prolong* N3 (immobilisation OR immobilization)) ) OR TI (restricted N1 activit*) OR AB (restricted N1 activit*) OR TI (chronic N2 limitation N2 activity) OR AB (chronic N2 limitation N2 activity) | 4,314 |
| S4 | (MH "Bed Rest") | 1,356 |
| S3 | S1 OR S2 | 987,713 |
| S2 | TI ( ((old N1 age) or (old* N2 (people or person* or adult* or men or women or male? or female?)) or elder? or elderly or "over 60*" or "over 65*" or "over 70*" or "over 75*" or "over 80*" or "over 85*" or "over 90*" or "over 95*" or sexagenarian* or septagenarian* or octogenarian* or nonogenarian*) ) OR AB ( ((old N1 age) or (old* N2 (people or person* or adult* or men or women or male? or female?)) or elder? or elderly or "over 60*" or "over 65*" or "over 70*" or "over 75*" or "over 80*" or "over 85*" or "over 90*" or "over 95*" or sexagenarian* or septagenarian* or octogenarian* or nonogenarian*) ) | 252,575 |
| S1 | (MH "Aged+") | 900,999 |
| Cinahl | | |

| Web of Science | |
| --- | --- |
| 1 | TS=("old age" or (old* NEAR/2 (people or person* or adult* or men or women or male* or female*)) or elder* or "over 60*" or "over 65*" or "over 70*" or "over 75*" or "over 80*" or "over 85*" or "over 90*" or "over 95*" or sexagenarian* or septagenarian* or octogenarian* or nonogenarian*) |
| 2 | TS=("bed rest" OR bedrest OR (bed NEAR/5 (confine* OR immobil*)) OR bedridden OR "bed ridden") or TI=(immobilisation OR immobilization) or TS=(prolong* NEAR/3 (immobilisation OR immobilization)) or TS="restricted activit*" OR TS=(chronic NEAR/2 "limitation of activity") |
| 3 | TS=(cognitive NEAR/2 (decline or status or capacity or recovery or outcome*)) or TS=(mental* NEAR/3 (decline* or challenge*)) or TS=(mortality or death* or fatalit* or "fatal outcome*") or TS=(hospitalization*or hospitalisation* or (length NEAR/2 stay) or ((patient* or hospital) NEAR/2 (admission* or admitted))) |
| 4 | 1 and 2 and 3 |

| ASSIA | |
| --- | --- |
| S1 | noft(("old age" or (old* NEAR/2 (people or person* or adult* or men or women or male* or female*)) or elder* or "over 60*" or "over 65*" or "over 70*" or "over 75*" or "over 80*" or "over 85*" or "over 90*" or "over 95*" or sexagenarian* or septagenarian* or octogenarian* or nonogenarian*) ) |
| S2 | noft(("bed rest" OR bedrest OR (bed NEAR/5 (confine* OR immobil*)) OR bedridden OR "bed ridden") ) OR ti((immobilisation OR immobilization) ) OR noft((prolong* NEAR/3 (immobilisation OR immobilization)) ) OR noft("restricted activit*" OR (chronic NEAR/2 "limitation of activity") ) |
| S3 | S1 AND S2 |

#
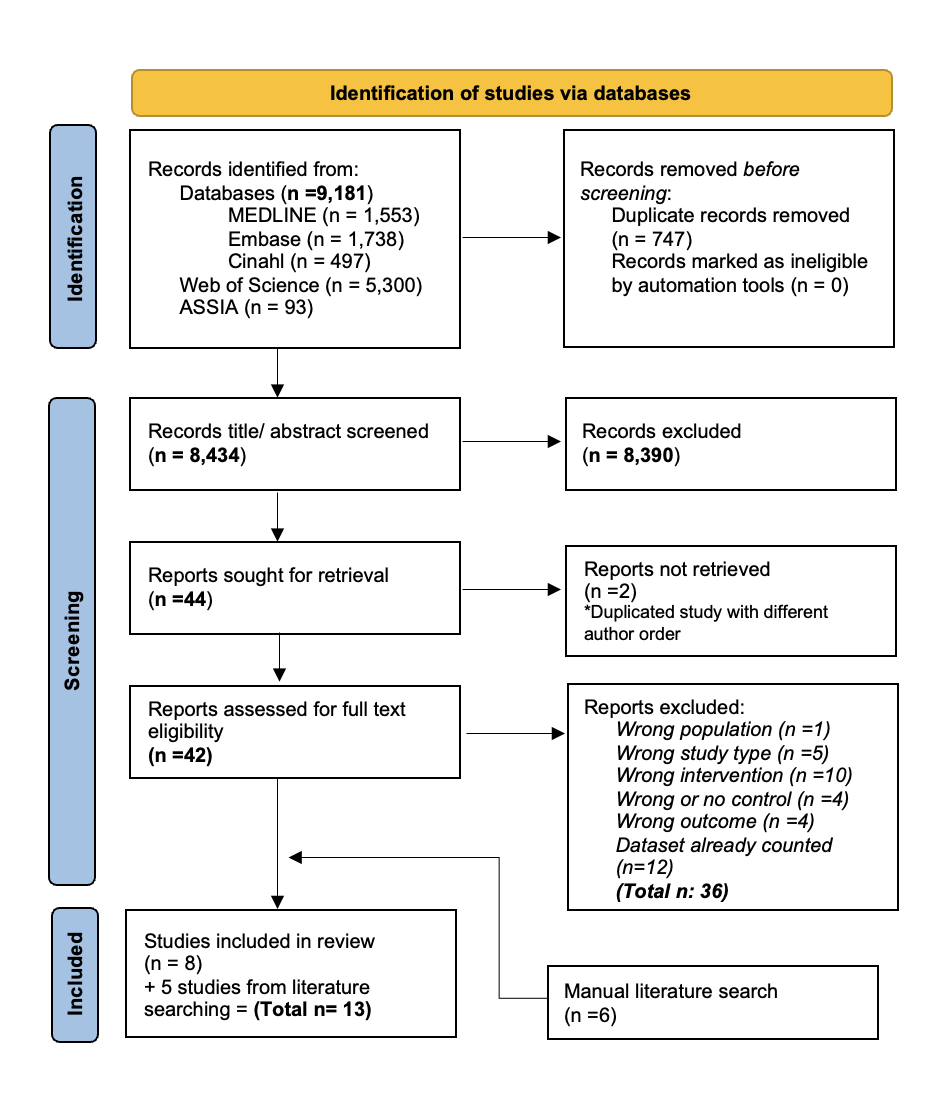
Appendix B: Prisma Diagram of included studies

**Figure B1: Prisma diagram** representing the number of studies identified in the initial search, and subsequent screening stages. This figure demonstrates how many studies were identified in each database, and how many studies were excluded at the different stages, the title and abstract screening, and then full text screening, stating why they were excluded. The total number of studies taken forward was 11.

# Appendix C: Meta-analysis sensitivity analysis

A sensitivity analysis was conducted using less conservative measures of restricted activity. This was to assess how robust the measures were.

The changes were:

Short follow-up

- Paloma, 2000, changed to >10 hours in bed (from >16 hours)
- Paloma, 2000, changed to partially dependent and dependent (from dependent)

Long follow-up

- Ginsberg, 1999, changed to >1 day in bed per fortnight (from >6 days)
- Brill, 1997, changed to unable to perform major activity and limited in kind or amount of major activity (from unable to perform major activity)
- Carey, 2008, changed to partially dependent and dependent (from dependent)


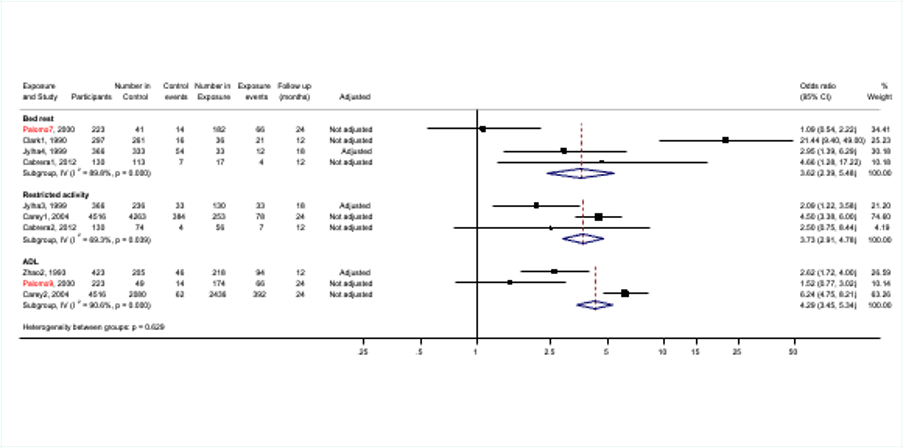


**Figure C1: Sensitivity analysis of restricted activity and mortality, with a short follow up (≤24)**. Analysis is sub-grouped by the type of restricted activity (bed rest, restricted movement, and activities of daily living (ADL) dependency. Square markers indicate the point estimate of the effect size within the different studies, with the whiskers indicating the confidence intervals. The size of the box correlates to the inverse variance of the effect estimate, which indicates the weight given to the study in the pooled analyses. The diamond markers indicate the pooled effect estimate across sub-groups. Superscript denotes the control for each study.


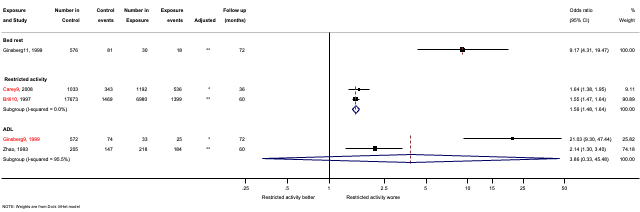


**Figure C2: Sensitivity analysis of restricted activity and mortality, with a long follow up (≥25)**. Analysis is sub-grouped by the type of restricted activity (bed rest, restricted movement, and activities of daily living (ADL) dependency. Square markers indicate the point estimate of the effect size within the different studies, with the whiskers indicating the confidence intervals. The size of the box correlates to the inverse variance of the effect estimate, which indicates the weight given to the study in the pooled analyses. The diamond markers indicate the pooled effect estimate across sub-groups. Superscript denotes the control for each study.

*= Not adjusted

**= Adjusted

^1^ No cut-down in usual activity

^2^Independent for ADL, or walking

^3^Spending day moving around

^4^ Spending day sitting or moving around

^5^Less than the defined cut off (16 hours) hours in bed

^6^Less than the defined cut off (6 days per fortnight) bed rest

^7^Less than the defined cut off (10 hours) hours in bed

^9^Independent & partially dependent for ADL, or walking

^10^ No cut-down, or limited cut down in usual activity

^11^Less than the defined cut off (1 day per fortnight) bed rest

# Appendix D: Evaluation of the different methods of restricted activity

Table D1: Table evaluating the different measures of restricted activity. SA= sensitivity analysis.

| **Author and year** | **Restricted activity group (restricted movement, bed rest, ADL general)** | **Method of capturing restricted activity** | **How was it captured** | **Rationale (e.g. how did they come up with this measure)** | **Other analysis from the same study? (including sensitivity analysis (SA))** |
| --- | --- | --- | --- | --- | --- |
| Brill, 1997 | Restricted movement | Data subjects participated in a National Health Interview Survey (NHIS) over a period of 5 years. Respondents were asked about activity limitation. For women aged 65-69, the major activity was working or keeping house, for those aged >70, it was the ability to live independently. If activity limitation was caused by a chronic condition, respondents were classed as limited in activity. The NHIS categorised respondents into 1 of 4 groups, dependant on how they perform the major activity. The groups were: unable to perform the major activity, limited in the amount or kind of major activity, limited but not in the major activity, and not limited. | Household interview survey conducted by an interviewer. | Data from the NHIS. The framework includes a different major activity for each age groups, 18-69 and 70 or over. The rationale is that as individuals over 70 years old daily activities tend more towards personal and household care than work related activities. It has been framed as a fairly general activity. | SA: Looked at limited but not in major activity (a broader definition)  Compared to the original analysis of unable to perform major activity. |
| Clark, 1990 | Bed rest | Data from participants living in the intermediate-care facility (ICF) of a nursing home. Those classed as ‘taking to bed’ experienced a rapid decline in independence in mobility. Episodes of rapid decline had to last at least 3 days to meet this definition, or residents who had gone to hospital and had been discharged back to the nursing home in a bed-bound state. Residents that experienced decline and then had been transferred to a skilled nursing facility (SNF) were also included, as ‘taking to bed’. Once participants had taken to bed, periodic nursing assessments were implemented to monitor changes in ability to perform activities of daily living. Progressive notes were reviewed to validate changes noted during monitoring. | Supervision from nursing home staff | ‘Taking to bed’ is a broad measure to encompass the 3 main instances when a participant undergoes rapid decline. On top of this, the researchers have a 3-day threshold to be appropriate to class someone under this measure. This helped to narrow the data subjects, and possibly provide more valuable data. | N/A |
| Gill, 2001 | Bed rest | Participants living in the community, part of the Precipitating Events Project. Baseline data was collected by trained research nurses using standard instruments, performing interviews and assessments, such as self-reporting, cognitive status assessment, and in person physical assessments. Follow-up data was collected through monthly telephone interviews using a standardised 4 step protocol. There were 2 questions related to restricted activity, identifying any functional decline and bed rest. Then participants were asked if they had any of the 24 prespecified problems in the interviewer’s framework. They were asked if any of these problems caused best rest or functional decline. Finally, they were asked if there were any other contributing factors. This research protocol underwent pilot testing and approval. | Data was collected through monthly telephone interviews, following a standardised protocol, to understand if participants had experienced bed rest or functional decline. | The research protocol underwent pilot testing, and was then approved by a university school of medicine institutional review board | N/A |
| Cabrera, 2012 | Restricted movement (use of walking aid)  Bed rest | Initial data evaluated functional capacity. Then a 1-year follow up visit carried out by different researchers at the data subjects’ homes, to take a measure of the participants functional capacity level, alongside other non-restricted activity measures. Participants were classified into 1 of 3 categories based on their mobility patterns. The 3 categories were: A) – those who could walk independently, B) – those who walked with the help of caregivers or with the use of walking aids, and C) – those who were bedridden or confined to a wheelchair. A statistics program stored and analysed the data, and the chi-square test was used to compare the different proportions and frequencies in the 3 functional groups (A, B, and C). | Initial at-home visits were conducted by multidisciplinary teams of trained researchers, and then a 1-year follow up home visit was undertaken by a different group of researchers for each data subject. | The study design gained ethical approval from a research committee within a university hospital in Brazil. The research protocol was also relayed to and agreed with the participants, who were also invited to give consent. Only 3 functional groups were used, to allow for a potentially large proportion of data subjects within each group. The 3 groups were distinct enough that data subjects could be easily classified, during the initial home visit or during the 1-year follow up. | Groups B)- those who walked with the help of caregivers or with the use of walking aids was used for restricted movement exposer  Group C) – those who were bedridden or confined to a wheelchair was used for bed ridden exposure |
| Zhao, 1993 | ADL General | Data was collected in interviews during home visits. The interviewers were all members of senior citizens’ clubs in Yao City, with financial help for the project coming from the local government. The subjects were interviewed using a set questionnaire, with 4 separate questions for 4 individual ADLs. These were eating, bathing, dressing and walking. Each ADL was scored from 1 to 3. One meant the ADL was possible without help. Two meant the ADL was possible with some help, and a 3 meant the participant was totally dependent on help for the ADL. The scores were then summed up, and general ADL level split into 2 categories, ‘high’ and ‘low’. Any data subject with a summed-up score of 7 or more were categorised as ‘low’, and a score of 6 or less was categorised as ‘high’. | These data were captured in a home setting, by a group of interviewers. The interviewers would attend the data subjects’ homes with a set questionnaire. | Following the Katz-index ([39](#_ENREF_39)) for independence. The ADL measure was created by taking 4 ADL activities and presenting each one on a 3 point scale. The 4 activities are broad enough to provide a general view of ADL. Based on the sum of individual scores for each of the 4 activities, a high/low category could be chosen for each participant. | N/A |
| Carey, 2008 | ADL General  Restricted movement | Patients were enrolled in the PACE (Program of All-Inclusive Care for the Elderly) program. A geriatric assessment was done by the PACE interdisciplinary team on patients at enrolment. The initial assessment was used as a zero-point, with follow ups over varying periods of time. The team used a standard protocol to assess patients. When it came to measuring functional status, 6 ADLs were assessed. Participants were categorised as independent, partially dependent, or fully dependent in each ADL. Partially dependent meant that the patient only required assistance some of the time, or with some of the ADL. Fully dependent meant the patient required assistance for the entire activity all of the time.  The ADL looking at walking was used independently for restricted movement. | Data was gathered by interdisciplinary teams using DataPACE (holds data on PACE patients). Sources of data included patients, caregivers, nurses, social workers and physicians. Data were collected initially as patients enrolled, and then continued as they were followed in the program. | Six basic ADLs (alongside 8 IADL). Covering a broad spectrum of ADLs helped form a more holistic understanding of each patient’s functional status. | ADL were also broken down into  Walking (used in restricted movement group)  Transferring  Dressing |
| Jylha, 1999 | Restricted movement (sitting and in bed)  Bed rest | Self-reported questionnaire consisted of 11 questions, in accessible font. One question was centred around which physical position each patient tends to spend most of their day. When asking how patients mainly spend their day, the three options were: ‘On feet’, ‘Sitting’ or ‘In Bed’. | A self-administered survey was sent to patients to complete, detailing self-reported indicators of functional status. | TA simple three-way question on how patients mainly spend their day, in relation to physical position, is easy for patients to recognise and report on themselves. | Sitting and in bed combined for restricted movement  In bed was used for bed rest |
| Ginsberg, 2000 | ADL General  Bed rest | At home interviews were performed by an occupational therapist under the supervision of a medical steering team. The home interview collected functional data on ADL (and IADL). An overall ADL score was given using the 6 dimensions from the Katz ADL index: bathing, dressing, toileting, transferring, eating, and continence. When independent in 5 or 6 of the dimensions the participant was classed as independent, and dependent when the score was lower.  Questions also captured data on bedridden days. Participants were classified as bedridden if they had spent at least 6 days, or 1 day, during the fortnight before the interview in bed. | Interviews in the home residence of the participants with almost all (95%) interviews undertaken by a single occupational therapist, with medical steering oversight. | The researchers utilised Katz index for ADL([39](#_ENREF_39)). The Katz index is well cited and well known.  Six days, and 1 day of the fortnight prior to the interview in bed. This captures a range between limited bed rest (>1 day), to significant bed rest (>6 days). | ADL data used for general ADL group  Bed days data used for bed rest group  >6 days used in initial analysis  SA: >1 day in bed was used in sensitivity analysis |
| Palomo, 2000 | Bed rest (>16 hours in bed)  ADL General | Participants came from a group of 16 family doctors. The group of doctors measured patients once at the start of the study, and then again 2 years after. The doctors would ascertain the ‘daily hours spent in bed’ status of each patient. This could be 1 of 3 options, ‘less than 10 hours’, ’10-16 hours’, and ‘more than 16 hours’.  The doctors would ascertain the status of each patient and score them against a criteria of ADL measures. The criteria of ADL used was the Katz index. Running each patient through the Katz index produced status for each patient, either ‘independent’, ‘partially dependent’, or ‘fully dependent’. | Data was collected by the family doctors. They assessed patients. The study was carried out at the home locations of the patients. | The range of hours in bed allowed patients to be divided into 3 different groups.  Followed Katz-index ([39](#_ENREF_39)) for independence, an established matrix. | SA: Initially looked at >16 hours in bed, and then at >10 hours in bed for sensitivity analysis  SA: Initially looked at fully dependent, and then partially dependent for sensitivity analysis |
| Carey, 2004 | ADL General  Restricted movement (Walking)  Transferring  Dressing | Part of the Asset and Health Dynamics Among the Oldest Old (AHEAD) study. Data were collected through interviews. Capturing activity was done through 6 ADL activities and scored, through self-reporting. The 6 ADLs assessed were eating, toileting, bathing, dressing, transferring, and walking across a room. Participants were classed on a 2-point scale, either independent or dependent, in relation to each ADL activity.  This was done as a general overall score, or focusing on the ADL activity for walking across a room, transferring, or dressing. | The participants self-reported on measures of functional status during interviews. In some cases, proxy interviews were used, such as if the interviewer deemed the data subject too impaired or ill, or when a data subject refused a direct interview but agreed to a proxy interview. | The researchers chose measures that could be obtained by the patient alone, using an interview framework that is easy to use. A wide range of ADL measures were used, to create an index that could discriminate between older people at varying risks. | ADL general was measured using an overall ADL score  Walking (used in restricted movement group)  Transferring  Dressing |
| Hardy, 2011 | Restricted movement | Self-reported ability to walk ¼ mile: “How much difficulty do you have walking a quarter of a mile—that is, about 2 or 3 blocks?” Participants rated their ability on the following scale: no difficulty at all, a little difficulty, some difficulty, a lot of difficulty, or not able to do it. | Interviewer goes to participants house 3 times a year. | Walking 1/4 mile has been proposed as an indicator of mobility | N/A |
| Rajan, 2013 | ADL General | The ADL disability measure focused on the ability to perform six essential self-care tasks: bathing, dressing, eating, showering, toileting, and getting out of bed to chair.  Responses contained three choices: “no help,” “help,” or “unable to do.” If the study participant could perform the activity with “no help,” questions were coded as “0” and the questions with choices of “help” or “unable to do” were coded as “1.” A summary score was computed by adding the scores on the six tasks and it ranged from 0 to 6. | Self-reported through interviews in participants homes. | Widely used measure of self-care tasks | N/A |
| Rajan, 2015 | Restricted movement | The first measure is based on the work by Rosow and Breslau, focused on tasks that require a certain degree of strength and mobility. The questions include “Are you able to do heavy work around the house, like washing windows, walls, or floors without help?”, “Are you able to walk up and down stairs to the second floor without help?”, and “Are you able to walk half a mile without help?” Each positive report was coded as “1” and added across the items to form a summary score (range, 0–3). The second measure is based on the work by Nagi, and evaluated basic upper- and lower-extremity functions. The Nagi measure of functional limitations is composed of the following questions, “How much difficulty, if any, do you have pulling or pushing large objects like a living room chair?”, “What about stooping, crouching, or kneeling?”, “Lifting or carrying weights over 10 pounds, like a very heavy bag of groceries?”, “Reaching or extending arms above shoulder level?”, and “Either writing or handling or fingering small objects?” Each of the 5 items is scored according to degree of difficulty, with reports of no or a little difficulty coded as 0, and reports of some or a lot of difficulty, or just unable to do, coded as 1. A summary score was created by adding responses to the individual items producing a score that ranged from 0 to 5. The authors created a combined Rosow-Breslau/Nagi measure based on 3-point Rosow-Breslau and 5-point Nagi measures (range: 0 – 8). | Self-reported through interviews in participants homes. | Within the overall disablement framework, limitations in basic, individual upper- or lower-extremity functions tend to occur earlier in the disability process than limitations in more complex tasks or functions captured by IADL and ADL limitations. | N/A |
